# Lilac and honeysuckle phenology data 1956–2014

**DOI:** 10.1038/sdata.2015.38

**Published:** 2015-07-21

**Authors:** Alyssa H. Rosemartin, Ellen G. Denny, Jake F. Weltzin, R. Lee Marsh, Bruce E. Wilson, Hamed Mehdipoor, Raul Zurita-Milla, Mark D. Schwartz

**Affiliations:** 1 USA National Phenology Network, National Coordinating Office, Tucson, AZ 85721, USA; 2 School of Natural Resources and the Environment, University of Arizona, Tucson, AZ, USA; 3 U.S. Geological Survey, Tucson, AZ 85721, USA; 4 Oak Ridge National Laboratory, Oak Ridge, TN 37830, USA; 5 Department of Geo-Information Processing, Faculty of Geo-Information Science and Earth Observation (ITC), University of Twente, Enschede 7500, The Netherlands; 6 Department of Geography, University of Wisconsin-Milwaukee, Milwaukee, WI 53211, USA

**Keywords:** Climate-change impacts, Flowering, Plant ecology

## Abstract

The dataset is comprised of leafing and flowering data collected across the continental United States from 1956 to 2014 for purple common lilac (*Syringa vulgaris*), a cloned lilac cultivar *(S. x chinensis* ‘Red Rothomagensis’) and two cloned honeysuckle cultivars (*Lonicera tatarica* ‘Arnold Red’ and *L. korolkowii* ‘Zabeli’). Applications of this observational dataset range from detecting regional weather patterns to understanding the impacts of global climate change on the onset of spring at the national scale. While minor changes in methods have occurred over time, and some documentation is lacking, outlier analyses identified fewer than 3% of records as unusually early or late. Lilac and honeysuckle phenology data have proven robust in both model development and climatic research.

## Background & Summary

Although phenology is now understood to be a key indicator of climate change impacts^1–3^, large-scale, coordinated phenological monitoring of lilac and honeysuckle—which respond predictably to air temperature and accumulated heat in a regionally coherent pattern—was initiated in the United States to supplement the use of weather observations in agricultural forecasts. In the western United States, monitoring of purple common lilac (*Syringa vulgaris*) was initiated in the late 1950's, and monitoring of two cloned honeysuckle cultivars (*Lonicera tatarica* 'Arnold Red' and *L. korolkowii* 'Zabeli') was initiated in the late 1960s^4^. The effort was replicated in the eastern US in the early 1960’s, but included a cloned lilac cultivar *(S. x chinensis* ‘Red Rothomagensis’) instead of the common lilac^3^.

The western program ended in the mid-1990s, except for a few dozen sites reactivated in 1997 (these data are also available at http://meteora.ucsd.edu/cap/lilac.html#Clim)^5^. The eastern network was terminated in 1986, re-initiated in 1988 and then expanded into a broader nationwide online phenology monitoring effort in 2009 (ref. 3).

The resulting dataset has been used for applications far beyond the original vision, from understanding vegetation feedbacks to climate^6^, to measuring large-scale variations plant climatic adaptation variations^7^. The Extended Spring Indices^8–10^, a set of bioclimatic models based on these lilac and honeysuckle data, have been used to calibrate remote sensing imagery^11^ and to advance our understanding of the effects of climate variability and change on spatial and temporal variations in spring onset in the U.S.^12–14^.

The observational dataset is unique in both its geographic and temporal coverage, and has considerable potential to support additional research and applications. Beginning in 2009, data collection on common and cloned lilacs continued alongside new data collection for hundreds of native species following very similar protocols^15^. The deep temporal record of the lilac and honeysuckle dataset has the potential to extend our understanding of trends among native species^9^. Long-term, continental-scale datasets are both rare and critically important to understand the causes and consequences of changing phenologies among cloned plants, ornamentals and native species^16–18^.

## Methods

In the historic Eastern^19^ and Western^20^ programs, participants were directed to plant new lilac or honeysuckle clones, and/or to observe established common purple lilac shrubs in unshaded, flat, convenient locations, away from roads and away from microclimatic pockets (e.g., cold air drainages). A single clone line for lilacs (*Syringa x chinensis* ‘Red Rothomagensis’) and two clone lines for honeysuckles (*Lonicera tatarica* ‘Arnold Red’ and *L. korolkowii* ‘Zabeli’) were planted and monitored throughout the period of record. Observers were asked to record on paper cards the dates that each of five phenological events, such as first leaf or full bloom, occurred on each of their selected or planted lilac or honeysuckle individuals (Table 1). Early program coordinators noted that in some cases all individuals of a species at a site were aggregated for reporting (i.e., the observer reported a single date representing an ‘average’ for all individuals at the site for each phenological event); however, there is no documentation to indicate for which sites this occurred.

**Figure 1:**
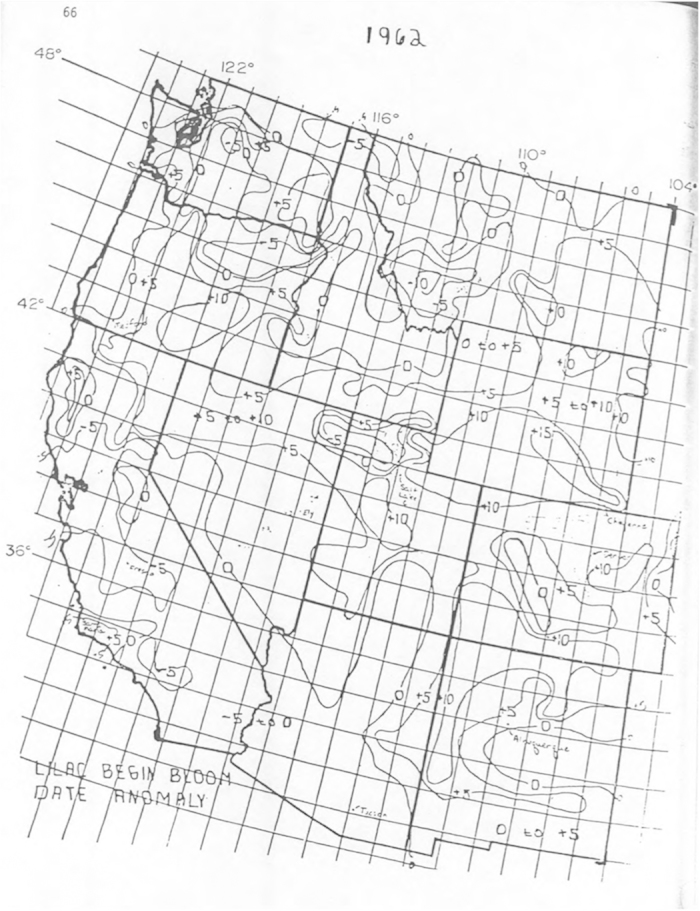
Lilac Begin Bloom Date Anomaly 1962. Isobars show anomaly, in days, relative to 1958–61 average first bloom date for the Western United States. Reproduced from Caprio *et al.*^21^.

**Figure 2:**
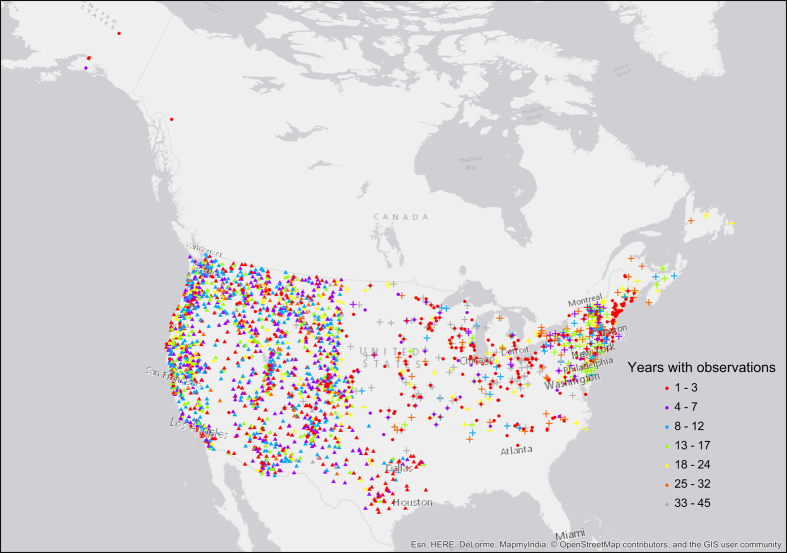
Data Collection Locations. Number of years with at least one observation recorded, for each site in the lilac and honeysuckle leafing and flowering dataset. Triangles indicate sites in the Western program, crosses indicate sites in the Eastern program and circles indicate sites in *Nature’s Notebook*.

**Figure 3:**
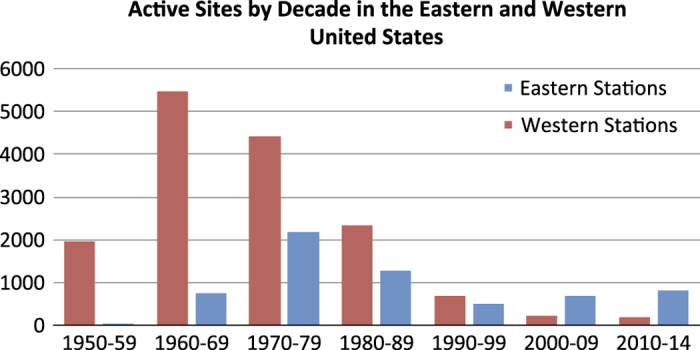
Observation Activity by Decade. Number of sites with at least one observation record, for each decade of the dataset, separated into the Eastern and Western programs (using −103 degrees longitude).

After all five phenological events had occurred for the year, observers mailed the cards to the program coordinators. Data were collated on paper by program coordinators, and isobar maps of spring arrival were developed and shared with observers (e.g., Fig. 1)^21,22^. The duration of record at each site is mapped in Fig. 2. The Western and Eastern programs differed in number of active sites (those with at least one observation record) by decade, as shown in Fig. 3.

The data were digitized and curated by Mark D. Schwartz, University of Wisconsin—Milwaukee, until the establishment of the USA National Phenology Network (USA-NPN) and the launch of its broader native plant monitoring program, *Nature’s Notebook* ^3,23^. The remaining active lilac and honeysuckle observers and their observation sites were incorporated into this program in 2009.

In 2009, the lilac and honeysuckle protocols were changed from their original event-based format (e.g., ‘What was the date of first bloom?’) to a status-based format (e.g., ‘Do you see open flowers today?’) to be consistent with the new USA-NPN plant protocols^15^. The rationale for this change in approach is that recording of the phenophase status on each day that the plant is observed enables detection of repeated phenological events within a single year (e.g., a second flush of breaking leaf buds after a killing frost), as well as calculation of the uncertainty in the date of phenological events (e.g., open flowers were first observed on April 6, but the plant had not been checked since April 3)^15^. Definitions provided to observers for the five leaf and flower phenophases, along with their changes over time between 1956 and 2014, are in Table 1.

*Nature’s Notebook* observers are still encouraged to plant and observe cloned and common lilacs as part of a campaign (https://www.usanpn.org/nn/campaigns), with electronic messages that provide real-time feedback on the progression of spring, information-rich species profile pages, and digital merit badges for tracking lilacs^23^. The cloned honeysuckle cultivars, however, are considered invasive and their planting is no longer encouraged.

## Data Records

The lilac and honeysuckle phenological dataset is an Excel workbook, stored in the Dryad Digital Repository (Data Citation 1) and USGS ScienceBase (Data Citation 2). The first tab (observation_data) contains 116,662 records for the period 1956 to 2014 across the United States. Each record includes a unique identifier, a site identifier and geographical information (latitude, longitude and elevation), species name, individual plant identifier, phenophase identifier and description, date of onset for each phenophase, and quality control flags. The second tab (site_data) details the number of years in record, and years missing, by site.

For observational data prior to 2009, phenophase onset dates (‘events’) were the only records submitted for each of the five phenophases on each individual plant in a given year. Beginning in 2009, status records could be submitted for each phenophase on each individual plant^15^. For temporal consistency in this dataset, we converted status data for the period 2009–2014 to onset dates by using the date of the first ‘Yes’ record of the calendar year for each phenophase on each individual plant. Where one or more ‘No’ records precede this first ‘Yes’ record, the number of days since the last prior ‘No’ is also provided, from which the uncertainty in the onset date can be quantified. Because this conversion can only be applied to status data, this feature is available for roughly 5% of the records in the dataset.

Although 2009 marked significant changes to the program (including the conversion from a paper to digital database, and the conversion from ‘event-based’ to ‘status-based’ monitoring) the meaning of each phenophase definition remained unchanged from the Eastern program definitions, in terms of pinpointing an onset date. However, there are some differences to note between the Eastern and Western program definitions for a few phenophases. ‘First leaf’ was defined by occurrence in a single location on the plant in both programs until 2005, when it was changed to three locations in the Eastern program only. ‘First bloom’ was defined by occurrence of a single open flower on the plant in the Western program, and, In the Eastern program, by the ‘date when at least 50% of the flower clusters have at least one open flower’ for lilacs and the ‘date when about 5% of the flowers are open’ for honeysuckle. Although the differences in ‘First leaf’ definitions probably represent an insignificant difference in reported onset date, the differences in ‘First bloom’ definitions could potentially represent a few days difference in reporting onset of the phenological event.

After 2009, observers were also able to provide additional information, including the absences of a phenophase prior to onset date (preceding ‘No’ records) and dates of phenophase presence after onset (subsequent ‘Yes’ records; e.g., indicating that open flowers were still present on the plant), as well as ancillary information about individual plants (e.g., shade status) and sites (e.g., degree of development). Descriptions of ancillary data are described at http://www.usanpn.org/files/shared/files/USA-NPN_suppl_info_tech_info_sheet_v1.0.pdf. Ancillary data and raw status data (for the period 2009–2014) are available at https://www.usanpn.org/results/data.

Dataset identifiers are also provided to distinguish data from the Eastern, Western and *Nature’s Notebook* sets. The geographic division between the Eastern and Western programs falls along the Great Plain states, with the Dakotas, Nebraska, Kansas and Oklahoma in the Eastern program, and Texas in the Western program.

## Technical Validation

**Figure 4:**
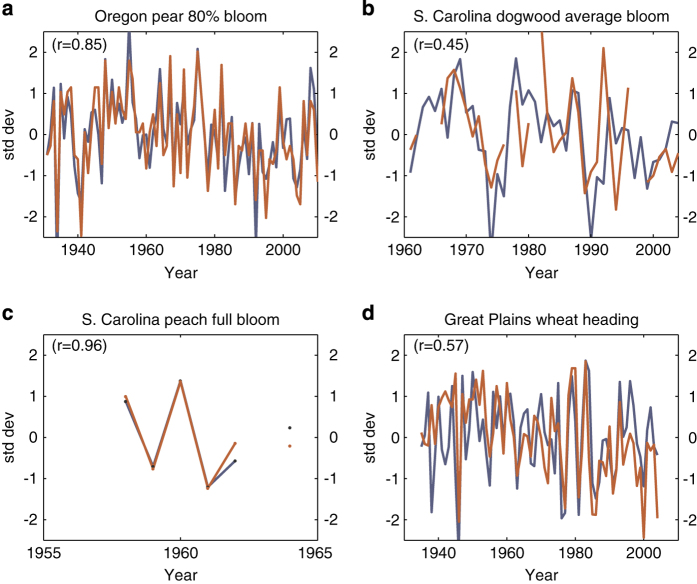
Comparison of SI-x to Other Species. Comparison of SI-x first bloom date (blue) with three crops and one native species phenological time series (red). All are displayed as z-scores (standard deviation units) for better visual comparison, and the Pearson's correlation for each pair is shown in the upper left corner. (**a**) Average SI-x first bloom dates across the state of Oregon (from 23 weather station sites) and anjou pear 80% bloom dates in the Rogue Valley of southwestern Oregon (Medford, Ashland, Grants Pass, near the California border) from 1931 to 2010 (G. Jones, unpublished pear data). (**b**) Average SI-x first bloom dates across the state of South Carolina (from 14 weather station sites) and average Cornus florida (dogwood) flowering time, derived from herbaria records, partial years from 1961 to 2007 (I. Park, unpublished dogwood data, complied using methodology in Park, 2012). (**c**) Average Si-x first bloom dates (as in Fig. 4(b)) and average peach full bloom dates among three varieties (Dixired, Elberta, and Red Haven) at two station sites from 1958 to 1962, and 1964 (peach data from Schwartz *et al.*^8^). (**d**) Average SI-x first bloom dates in the United States region north of 35°N latitude and between 90° and 100°W longitude (from 153 weather station sites) and winter wheat (Kharkof cultivar) heading dates at six sites from 1935 to 2004 (wheat data from Hu *et al.*, 2005). Reproduced from Schwartz *et al.*^9^.

While information on monitoring frequency, observers and data management practices prior to 2009 is not available, the dataset is otherwise well-documented and has been proven reliable in several publications. Early work with the dataset explored the relationship between biological and climatological spring^6,24,25^. Further demonstration of the utility and validity of the lilac and honeysuckle dataset is found in the Spring Indices^8^. These models, developed using the dataset presented here, predict lilac leafing and blooming dates based on the strong relationship between day of year, heat accumulation, number of high-energy synoptic events and the timing of these phenological events^8^. Recent work has extended these indices across the continental United States, using both weather station data^9^ and gridded climate products^26^, and found strong relationships between Spring Indices (SI) predictions and the timing of phenological events for native species and crops (Fig. 4)^9^.

Minor cleaning of the Eastern and Western program data was conducted by the authors in 2013, and included the removal of duplicate records (4 records), those with null location information (35 records), and those with phenophase onset dates reported in an implausible order (72 records). For the publication of this dataset, we additionally excluded onset dates where status records conflicted (21 records; i.e., a ‘Yes’ and a ‘No’ status were reported on the same day; these flagged records are available via download at https://www.usanpn.org/results/data).

In the field, under natural or managed ecosystems and for a variety of plant species, it is possible to have multiple onsets of the same phenophase on an individual plant in a calendar year (e.g., a second flush of breaking leaf buds after a killing frost, or a second round of flowering in the fall). These multiple onsets are detected in the status data when the first series of consecutive ‘Yes’ records is concluded with at least one ‘No’ record, then a subsequent ‘Yes’ record is present. The second onset date is the date of this subsequent ‘Yes’ record. A flag field (‘Multiple_FirstY’) in the data file indicates those cases where more than one onset for a phenophase occurs in a calendar year; this occurs in less than 1% of the records in the data file.

Occasionally, at sites where several observers monitor an individual plant, multiple onsets are calculated from the raw status data as a result of different observers ‘leapfrogging’ over each other with slightly different phenophase interpretations. For example, one observer might report a ‘Yes’ for ‘Open flowers’ when they judge conditions on individual lilac have *just* passed the threshold for occurrence. On a subsequent day, another observer might report ‘No’ if they judge conditions on the same individual have *not quite* passed the threshold for occurrence. If the first observer subsequently reports a status of ‘Yes’ there will appear to be two distinct onsets. Where these likely false onsets occur in the data (i.e., where associated ‘MultipleFirst_Y’ records are separated by very short time periods), it is reasonable to consider the first onset of the calendar year as the true onset, unless quality control flags indicate that the first onset is implausible. Groups are encouraged to resolve these issues and the raw status data can be explored for comments from observers, to confirm the occurrence of true multiple onsets within a calendar year.

We identified outliers in phenophase onset dates for individual plants, across the period of record, using, Tukey boxplots^27^. We flagged records with onset day of year values greater/less than 1.5 times the interquartile range for each individual. We excluded individual plants with less than 10 years of data, and used the first onset of the calendar year in cases where there were multiple first ‘Yes’ records. Of 67,068 tested records (57% of the dataset), 0.88% were flagged as unusually early (‘Individual_Outlier’ of −1 in the dataset) and 0.58% were flagged as unusually late (‘Individual_Outlier’ of 1).

We applied a more complex outlier analysis^28^ for the dataset as a whole, to identify unusually early or late phenological events, based on the location of the observed plant and the interannual variation in climatological conditions at that location. This analysis relies on the assumption that plant phenology is influenced by environmental conditions, and therefore the variability of phenological observations made under similar environmental conditions must be relatively small (given unknown variation in microclimate, or genetic variation among organisms).

To apply this quality-control check, we used DAYMET, which is the highest spatial resolution, gridded dataset freely available for the United States. Because DAYMET is only available since 1980 and was not yet available for 2014 at the time of this analysis, we were able to apply this check to 39,791 records (34% of the dataset); this quality check could be applied to earlier data if additional fine-scale climatological data become available. For each observation year and location where observations were made (including all multiple first ‘Yes’ dates), we calculated cumulative values from 1 January to phenophase onset date (expressed as day of year; DOY), for the following meteorological parameters: surface minimum and maximum temperatures, precipitation, humidity, shortwave radiation, snow water equivalent, and day length. Using the daily maximum and minimum temperatures, average temperatures were calculated and then summed up in a similar way. To this set of meteorological data, we added the latitude, longitude and elevation of each observation. Then, we applied the t-SNE dimensionality reduction algorithm^29^ to project the climatological and geographic variables into a two-dimensional space. This allows visualization and interpretation of the results. After that, we applied a model-based clustering^30^ to group the observations into sets made under similar environmental conditions. Finally, the Tukey boxplot^27^ was applied to highlight the outliers present in each of the clusters. These outliers (with values greater/less than 1.5 times the interquantile range of each cluster) are highlighted as inconsistent observations. We flagged 0.74% of the records as unusually early (‘Inconsistency_Flag’ of −1 in the dataset) and 1.35% of the records as unusually late (‘Inconsistency_Flag’ of 1). The impact of inclusion or exclusion of these observations has yet to be explored.

## Usage Notes

The raw status data used to produce this dataset, and information about the *Nature’s Notebook* sites, plants and observers is housed in the National Phenology Database and is available for download via the USA-NPN website (https://www.usanpn.org/results/data). Lilac observations submitted after 2014, as well as honeysuckle fruiting data from the Western program for the period 1968–2009, can also be downloaded from this website.

## Additional Information

**How to cite this article:** Rosemartin, A. H. *et al.* Lilac and honeysuckle phenology data 1956–2014. *Sci. Data* 2:150038 doi: 10.1038/sdata.2015.38 (2015).

## Supplementary Material



## Figures and Tables

**Table 1:** Western, Eastern and *Nature’s Notebook* phenophase definitions for the five phenological events or phenophases

	**Western Network**	**Eastern Network**	***Nature’s Notebook***
Reference	^20^	^19^	^15^
Dataset ID	8	7	Null (−9999), 3
Years	1956–2008	1961–2008	2009–2014
Leaf Phenophases for Lilac and Honeysuckle	First leaf (76): Date when first bud has leafed. A bud is in leaf on the date when you first find that the widest part of a newly emerging leaf has grown beyond the ends of its opening winter bud scales. The leaf is distinguished by its prominent mid-ribs and veins.	First leaf (76): Date when the widest part of the newly emerging leaf has grown beyond the ends of its opening winter bud scales. The leaf is distinguished by its prominent midrib and veins. *From 2005 to 2008, the definition specifies this must be occurring in at least three places on the plant.*	Breaking leaf buds (373): In at least 3 locations on the plant, a breaking leaf bud is visible. A leaf bud is considered ‘breaking’ once the widest part of the newly emerging leaf has grown beyond the ends of its opening winter bud scales, but before it has fully emerged to expose the leaf stalk (petiole) or leaf base. The leaf is distinguished by its prominent midrib and veins. *From 2009 to March 2011, ‘breaking leaf buds’ in this definition was referred to instead as ‘emerging leaves’.*
Leaf Phenophases for Lilac and Honeysuckle	Full leaf (75): Date when nearly all (at least 95%) of the actively growing buds have already leafed.	Full leaf (75): Date when nearly all (at least 95%) of the actively growing leaf buds have already leafed.	All leaf buds broken (374): For the whole plant, the widest part of a new leaf has emerged from virtually all (95–100%) of the actively growing leaf buds. *From 2009 to March 2011, this phenophase was referred to as ‘All leaves emerged’.*
Flower Phenophases for Lilac	First bloom (412): Date of opening of first bloom is the date when the first flower is fully open. The lilac flower cluster is really a grouping of many small individual flowers, so the date to record is the date when one of the small flowers in a cluster is fully open.	First bloom (77): Date when at least 50% of the flower clusters have at least one open flower. The lilac flower cluster is a grouping of many, small Individual flowers.	Open flowers (205): For the whole plant, at least half (50%) of the flower clusters have at least one open fresh flower. The lilac flower cluster is a grouping of many, small individual flowers.
Flower Phenophases for Lilac	Peak of full bloom (78): Date when nearly all of the flowers on the plant are open, but before any appreciable number of them have withered or dried up.	Full bloom (78): Date when 95% of the flower clusters no longer have any unopened flowers, but before many of the flowers have withered.	Full flowering (206): For the whole plant, virtually all (95–100%) of the flower clusters no longer have any unopened flowers, but many of the flowers are still fresh and have not withered.
Flower Phenophases for Lilac	End bloom (79): Date when nearly all (at least 95%) of the flowers have withered or dried up is the date when the floral display has ended, except possibly for several clusters on the bush.	End bloom (79): Date when at least 95% of the flowers have withered or dried up and the floral display has ended.	End of flowering (207): For the whole plant, virtually all (95–100%) of the flowers have withered or dried up and the floral display has ended.
Flower Phenophases for Honeysuckle	First bloom (428): Date of opening of first bloom is the date when the first flower is fully open.	First bloom (428): Date when about 5% of the flowers are open.	Open flowers (210): For the whole plant, at least 5% of the flowers are open and still fresh.
Flower Phenophases for Honeysuckle	Peak of full bloom (78): Date when nearly all of the flowers on the plant are open, but before any appreciable number of them have withered or dried up.	Full bloom (429): Date when 95% of the flowers are open, but before many have withered.	Full flowering (211): For the whole plant, virtually all (95–100%) of the flowers have opened, and many of the flowers are still fresh and have not withered.
Flower Phenophases for Honeysuckle	End bloom (416): Date when nearly all (at least 95%) of the flowers have withered or dried up is the date when the floral display has ended, except possibly for just a few branches.	End bloom (79): Date when at least 95% of the flowers have withered or dried up and the floral display has ended.	End of flowering (207): For the whole plant, virtually all (95–100%) of the flowers have withered or dried up and the floral display has ended.
Rows contain equivalent phenophase definitions for each program. Phenophase identifiers (‘Phenophase_ID’ field in the data file) are in parentheses. Where definitions have slight variations in meaning they are given different identifiers, however, equivalent definitions are unified by the ‘Phenophase_Group’ field in the data file. *Nature’s Notebook* data submitted through the online interface has a null Dataset ID, while data submitted via mobile app has a Dataset ID of 3.			
